# Book Notices and Reviews

**Published:** 1864-03

**Authors:** 


### BOOK NOTICES AND REVIEWS.

*The Nervous and Vascular Connection between the Mother and Fœtus in Utero.* By John O'Reilly, M. D., F. R. C. S., I. New York: Printed by Robert Craighead, 1864.

From believing that the ever varying mental conditions of the Mother, exercise a direct and controlling influence upon the nutrition and development of the Fœtus in Utero, and thus affording an explanation of the presence of "Marks" upon the surface, and abnormal developments of the various parts of the body of the Child, so frequently seen, the Profession passed to the other extreme, and held that these occurrences were accidental and entirely inexplicable. Now again the Physiologist is directing the Medical mind back, and by the advance in Anatomical and Physiological knowledge of the present day, endeavors to furnish the rationale of the occasion of these irregularities. Speaking of the influence of the maternal imagination in causing deformities and marks in the Fœtus, Dunglison says, "As a cause, therefore, the influence of the maternal imagination has been generally regarded as an inadmissible hypothesis. By many it has been esteemed ridiculous." *Phys.*, 2d *Ed.*, 1836. A late and popular work expresses the opposite opinion in the following language: "There is now little room for doubt that various deformities and deficiencies of the Fœtus, conformably with the popular belief, do really originate in certain cases from nervous impressions, such as disgust, fear, or anger, experienced by the Mother." *Dalton's Physiology*.

This pendulum movement of the Medical mind upon this subject, the author endeavors to bring to an equipoise, in the morceau before us, the latest addition to the literature upon the topic—the perusal of which has afforded us much pleasure. The subject is treated somewhat in the following order:—1st. That motion and pain are connected with the presence of nerves. 2d. That impressions made upon the cerebro-spinal system, are communicated and extend to the organic nervous system, through the inosculation of the nerves of the former with those of the latter. 3d. The following question is proposed, Is there a nervous connection between the Mother and Fœtus in Utero?

"It is a question, too, which has baffled the greatest Anatomists and Physiologists of the past and present generation to solve. Evidence of the truth of a connexion existing between the mother and fœtus in utero is continually presented, and, although the *vox populi* affirms the truth of such a connexion, judging from facts and the evidence of the senses, yet, strange to say, the medical profession has failed to give a satisfactory account of the phenomena presented in it, and ignores as well as repudiates the idea of there being any nervous connexion between the mother and fœtus in utero.

Mr. Quain says: "Now, as to the *sympathetic* nerve, so far from being in any way derived from the brain or spinal cord, it is produced independently of either, and *exists* notwithstanding the *absence of both*. It is found perfectly *formed* in *acephalous* infants, therefore *does not arise*, mediately or immediately, from the *brain;* neither can it be said to *receive roots* from the *spinal cord*, for it is known to exist *as early* in the fœtal state as *the cord* itself, and be fully *developed* even though the latter is *altogether* wanting. It appears that whilst the organs of vegetation and life are being formed, the sympathetic nerves are *produced concurrently* with them; and that as the growth of these proceeds from the *circumference* to the centre of the whole body, from its lateral parts to the median line, the *sympathetic nerves* also *conform* to the general law."—See Quain's *Elements of Anatomy*, p. 711.

Mr. Harrison, the celebrated anatomist, says, in speaking of the organic nerves which surround the aorta, "the latter accompany the common iliac artery to their division, and several filaments are prolonged around the internal and external iliac vessels."

These organic nerves are prolonged from the internal iliac artery along the umbilical arteries to the placenta, there inosculates with the corresponding class of nerves, which surround the uterine arteries.

4th. That there is a *direct* vascular connection between the mother and fœtus is *inferred*, from the *assumed* analogy between the placenta and liver—each organ having four sets of vessels appropriated to corresponding offices.

The circulation in these several vessels of the placenta, during gestation is manifestly powerfully affected by various states of the mind, by accident, etc. Proof—Hearing unexpected intelligence of an exciting character, produces abortion.

"Here the impression is conveyed from the filaments of the auditory nerve to the brain, spinal cord, sacral ganglia, connected with the roots of the spinal nerves, the hypogastric plexus, the nerves surrounding the arteries, distributed to the uterus, causing contraction of the muscular fibres of this organ and followed by the expulsion of the *ovum*."

Various circumstances may impress the nervous centres of the mother, be transmitted to the fœtus, occasion irregularity in the circulation of a part and defective nutrition, and thus produce nevi and deformities. The author's views are clearly stated—his arguments, if not demonstrative, are at least ingenious, his conclusions logical—and will well repay the time devoted to their study. Many cases are collated to illustrate the positions taken—none of which are more remarkable than an instance occurring in the practice of Prof. Miller, of Rush Medical College:

Mrs. W., in her third pregnancy, while passing to different parts of the city in following her vocation, ladies hair dressing, frequently met an individual in the streets, who was considerably deformed, and was severely affected with Chorea. The sight of this individual, with his fantastic and purposeless movements, his idiotic countenance, and distortion of body, produced a strong and repulsive impression upon her mind, so that she frequently expressed the fear that the effect would be unfavorable to the perfect development of her child. During the *progress of the labor* she manifested her anxiety upon this point. The labor, which took place March 26, 1863, was natural. The child, a female of the ordinary size, lived for a few minutes, and presented the following peculiarities:

On the back part of the head was a tumor the size of a large orange. It had hare-lip and cleft palate. The inferior extremities were of unequal length; both feet turned inwards (talipes varus); on each foot were six toes; on each hand five *fingers.* The specimen is preserved in the Museum of Rush Medical College.

---

*St. Louis Medical and Surgical Journal.—Bi-Monthly.* Edited by Profs. M. L. Linton, M. D., and F. W. White, A. M., M. D. New Series, Vol. 1., No. 1. Terms, $3,00 per annum.

After an absence of three years this valuable Medical Journal has again made its appearance on our table, looking as bright and neat as it was wont to do of yore. And its pages are so well filled with valuable matter that it will lose nothing by a comparison with its former self, or any other Journal in the country.

We congratulate the Editors on the favorable auspices under which they renew their editorial labors, and cordially welcome the *St. Louis Med. and Surg. Journal* to our exchange list.

---

*Notices Deferred.*—From want of space in the present number we are compelled to defer till our next issue, suitable notices of the following works:

*On Asthma, Its Pathology and Treatment.* By Henry Hyde Salter, M. D., F R. S., Fellow of the Royal College of Physicians, Assistant Physician to Charing Cross Hospital, and Lecturer on Physiology and Pathology at the Charing Cross Hospital Medical School. Philadelphia: Blanchard & Lea. 1864.

*Parish's Practical Pharmacy.* 3d Edition. By the same Publishers.

*Ellis' Medical Formulary.* New Edition.

*Dalton's Treatise on Human Physiology.* 3d Edition. By the same Publishers.

